# Assessment of left ventricular diastolic function after Transcatheter aortic valve implantation in aortic stenosis patients by echocardiographic according to different guidelines

**DOI:** 10.1186/s12947-020-0184-9

**Published:** 2020-01-21

**Authors:** Yao Guo, Minmin Sun, Haiyan Chen, Dehong Kong, Xianhong Shu, Cuizhen Pan

**Affiliations:** 1 Department of Echocardiography, Zhongshan Hospital of Fudan University, No180, Fenglin Road, Xuhui District, Shanghai, 200032 China; 2 Shanghai Institute of Cardiovascular Diseases, Shanghai Institute of Medical Imaging, No180, Fenglin Road, Xuhui District, Shanghai, 200032 China

**Keywords:** Transcatheter aortic valve implantation, Diastolic function, Transthoracic echocardiography, Doppler imaging

## Abstract

**Background:**

To evaluate the detailed dynamic change of left ventricular diastolic function (LVDF) by echocardiography in aortic stenosis (AS) patients receiving transcatheter aortic valve implantation (TAVI) and compare LVDF classification according to 2009 ASE/EAE and 2016 ASE/EACVI recommendations.

**Methods:**

Thirty-five AS patients receiving TAVI underwent echocardiography the day before operation (PRE), on the third day (3D), in the first-month (1 M) and the six-month (6 M) after TAVI. LVDF was analyzed using 2D and doppler imaging to get parameters including E/A, E/e’, isovolumic relaxation time (IVRT), deceleration time, LA area, LA volume index (LAVI) and systolic tricuspid regurgitation velocity (TR). LVDF classification was evaluated four times for each patient according to 2009 and 2016 recommendations respectively and the results were compared.

**Results:**

The decrease of IVRT and TR occurred immediately post surgery up to 1-month. Improvement of E/e’ occurred late from 3-day to 1-month. LA area and LAVI decreased continuously shortly after operation till 6-month. Forty-four percent (62/140) by 2009 recommendations were reclassified with different grades when using 2016 guidelines. Comparing PRE and 6 M, with 2009 guidelines, 19 patients improved 1 grade, 8 patients improved 2 grades; with 2016 guidelines, 9 patients improved 1 grade, 13 patients improved 2 grades, 1 patient improved 3 grades.

**Conclusions:**

The conventional 2D echocardiography could effectively reflect variation process of LVDF in AS patients after TAVI. For LVDD classification, obvious differences resulted by the 2009 and updated recommendations were found, and more patients can be regarded as benefiting from TAVI by 2016.

recommendations.

## Background

In an aging society, the morbidity of severe symptomatic aortic stenosis (AS) caused by retrogression or calcification steadily increases [1]^.^ Although surgical aortic valve replacement (SAVR) under extracorporeal circulation was considered as a major therapeutic method, approximately one-third of AS patients cannot undergo SAVR due to its’ high risk or contraindication [2]. Since Cribier et al. performed transcatheter aortic valve implantation (TAVI) for the first time in 2002 [3], TAVI has shown to be a feasible and effective therapeutic alternative for AS patients who could not perform SAVR [4, 5].

Conventional 2D transthoracic echocardiography (TTE) and Doppler imaging have been known as frequently-used methods with its noninvasiveness and convenience [6–9]. To our knowledge, most researches on TAVI just paid attention to diastolic improvement but ignored the detailed dynamic change which can provide useful information for clinical management. Importantly, for left ventricular diastolic dysfunction (LVDD) assessment, the difference in clinical application between 2009 ASE/ EAE and 2016 ASE/EACVI recommendations was still equivocal. Therefore, the main purpose of this study was to assess the echocardiographic LVDD grading after TAVI in AS patients and identify the differences between 2009 and 2016 algorithms.

## Materials and methods

### Patients

**Fig. 1:**
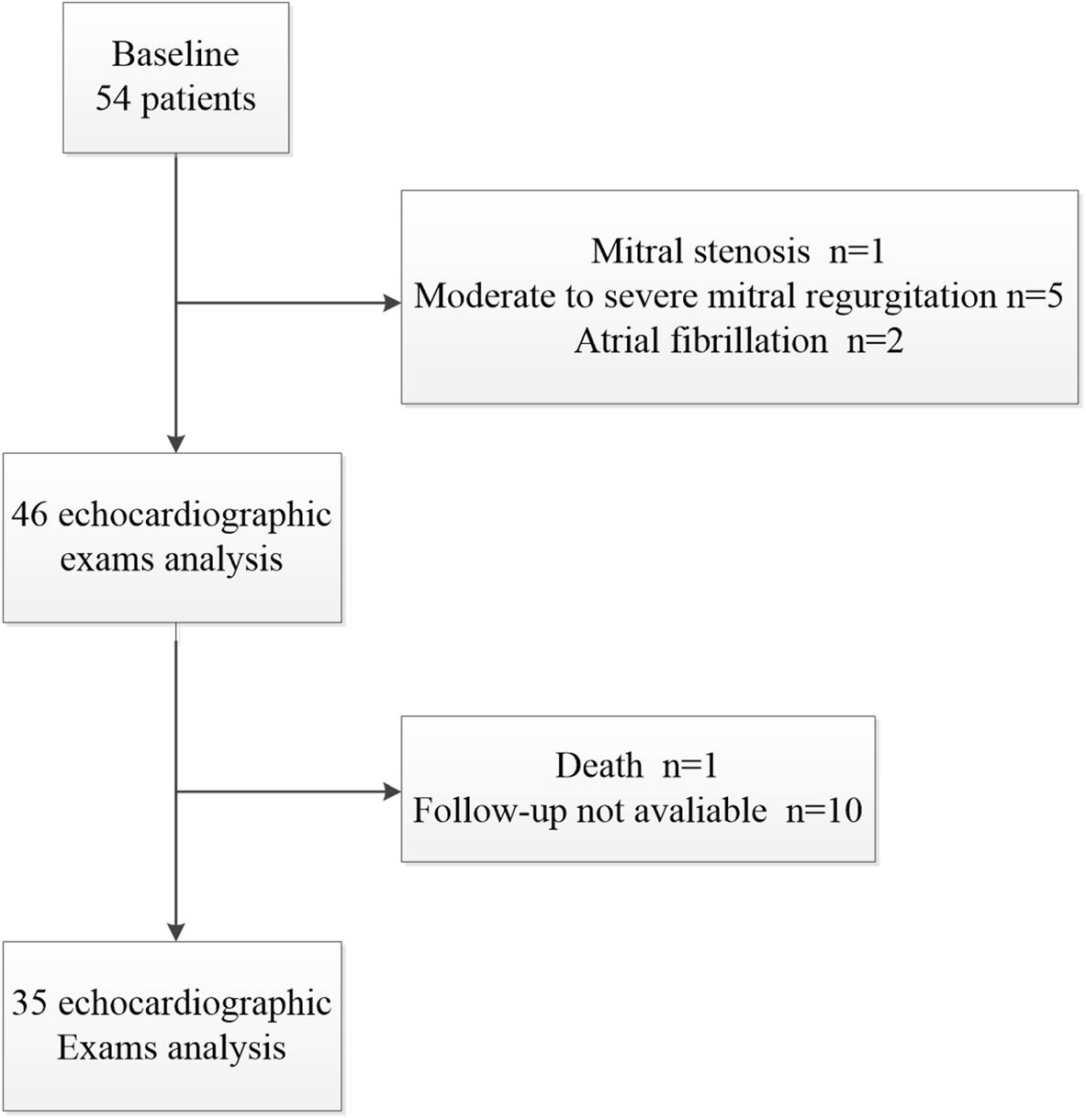
Diagram of the flow of participation

This study was approved by the institutional review board of our hospital, and the requirement for informed consent was waived. The diagnosis of severe AS was defined as a mean transaortic pressure gradient > 40 mmHg and an aortic valve area < 1 cm^2^ [10]. Fifty four patients with severe symptomatic AS who underwent TAVI between September 2013 and May 2017 were found by searching the clinical databases at our institution. Inclusion criteria were as follows: (1) patients who had severe aortic stenosis, (2) patients whose age > 70 (These patients had significant aortic valve calcification and were therefore suitable for self-expanding artificial aortic valves), (3) patients with sinus rhythm. Exclusion criteria: (1) patients with significant mitral valve disease, (2) patients with uncontrolled atrial fibrillation (including paroxysmal and persistent atrial fibrillation), (3) poor acoustic window and incomplete follow-up (Fig. 1). Specifically, One patient who was died 5 month later after TAVI because of the severe chronic obstructive pulmonary disease and respiratory failure was excluded. Ten patients did not have available echocardiographic images within 6 months, either lost to follow-up (*n* = 5) or poor acoustic windows (n = 5). Finally, there were a total of 35 patients available for analysis.

### Echocardiography

Comprehensive echocardiography was performed 1 day before TAVI (PRE) and on the third day (3D), in the first- (1 M), and six-month (6 M) after the procedure, using available equipment (iE33, Philips Medical Systems, N.A., Bothell, WA, USA) equipped with a broadband (1–5 MHz) S5–1 transducer. The images (standard 2D parasternal long-axis, short-axis images and apical 2-, 3-and 4-chamber views) were conducted in left lateral decubitus position. All measurement methods were according to the recommendations of American Society of Echocardiography (ASE) and European Association of Cardiovascular Imaging (EACVI) [11]. For each case, two readers (G.Y. and S.M.M. with 3 and 10 years of experience reading Echocardiographic exams, respectively) independently performed the analyses at the same workstation.

### Two dimensional echocardiography

In the parasternal long-axis view, 2D LV diameter (left ventricular end-diastolic/systolic diameter, interventricular septum thickness and posterior wall thickness) were obtained. The 2D LVEF was derived from the biplane Simpson method. LV mass (LVM) was calculated by the formula: 0.8 * {1.04 * [(IVST + LVEDD + PWT)^3^ - LVEDD^3^]} + 0.6 g. LA area and LA volume were calculated by biplane 2D Simpson method [12]. LA volume index (LAVI) was calculated by dividing the maximal LA volume by the body surface area (LAV/BSA). LA dilatation was defined as LAVI> 34 mL/m^2^ [13]. Peak and mean systolic transaortic gradients were calculated using the simplified Bernoulli equation [14].

### Doppler imaging

In the apical four-chamber view, the pulsed-wave (PW) Doppler sample volume was targeted at the tips of the mitral valve to measure the peak early (E) and late (A) diastolic transmitral filling velocities and E wave deceleration time (DT) on PW spectral Doppler. The peak early diastolic mitral annular velocities (e’) were obtained by placing the PW tissue Doppler imaging (TDI) sample volume on both the lateral and septal mitral annulus. Isovolumic relaxation time (IVRT) was assessed by placing PW spectral Doppler sample volume in LV outflow tract, displaying the curve and measuring the distances from the end of aortic outflow during systole to the onset of mitral inflow during diastole. In the same view, the continuous-wave (CW) Doppler sample volume was targeted at the tips of the tricuspid valve to assess systolic tricuspid regurgitation velocity (TR).

### Grading of LVDD

We assessed the LVDD grade in AS patients before and after TAVI by using 2009 ASE/ EAE and 2016 ASE/EACVI guidelines respectively [11, 15]. The comparison of echocardiographic parameters in two recommendations were showed in Table 1. According to the 2016 guidelines, LVDD grade should be determined by using two algorithms. In patients with normal EFs, the four recommended variables for identifying diastolic dysfunction and their cutoff values were (1) average E/E′ ratio > 14; (2) septal E′ < 7 cm/sec or lateral E′ < 10 cm/sec; (3) LA volume index > 34 mL/m^2^; (4) peak TR velocity > 2.8 m/sec. LVDF was normal if more than half of the available variables do not meet the cutoff values. The study was inconclusive if half of the parameters do not meet the cutoff values. If more than half of the available parameters met these cutoff values, the grade was assessed by the following algorithm.

**Table 1:** Comparison of echocardiographic parameters according to 2009 and 2016 left ventricular diastolic function recommendations

2009/2016 Parameters	2009 Parameters	2016 Parameters
Average E/e’	Valsalva E/A	E/A + E
Septal/Lateral e’	DT	TR
LAVI	Ar-A	LVEF
E/A		

*LAVI* left atria maximum volume index; *DT* deceleration time of E-wave; *TR* tricuspid regurgitation systolic peak velocity;

In patients with depressed EFs and in patients with normal EFs and myocardial disease, the grade was divided into multiple stages of severity: grade I (relaxation abnormality), grade II (pseudonormal), grade III (restrictive) and indeterminate. The main criteria for the grading severity were (1) if E/A ≤ 0.8 along with E ≤ 50 cm/s, the patients had Grade I LVDD; (2) if E/A ≥ 2 (DT was usually < 160 ms or normal), grade III LVDD was presented; (3) if E/A ratio ≤ 0.8 along with E > 50 cm/s, or 0.8 < E/A < 2, the additional parameters and cutoff values were (1) TR > 2.8 m/s; (2) average E/E’ > 14; (3) LAVI > 34 ml/m2. If all three parameters were available for interpretation and more than one of the variables met the cutoff values, the patients had grade II LVDD. If only one of three available variables met the cutoff value, grade I LVDD was presented. If only one parameter was available, grade of diastolic dysfunction should not be reported and likewise if there was discrepancy between the only two available parameters.

### Statistical analysis

Continuous variables were presented as the mean ± standard deviation, and categorical variables were presented as percentages. Normal distribution of continuous variables was tested using the Paired sample *t*-test and abnormal distribution of continuous variables using Wilcoxon signed-rank test. Interobserver reliability of the parameters were assessed using intraclass correlation coefficient (ICC) and Bland-Altman analysis. A value of 1.0 indicated perfect agreement; 0.81–0.99, almost perfect agreement; 0.61–0.80, substantial agreement; 0.41–0.60, moderate agreement; 0.21–0.40, fair agreement; and 0.20 or less, slight agreement. Differences with a *P*-value less than 0.05 were considered statistically significant. All statistical analyses were performed using statistical software (SPSS, Inc., Version 22.0, Chicago, IL, USA and MedCalc for Windows, Version15.8).

## Results

### Baseline clinical and echocardiographic findings

Baseline characteristics of enrolled patients were showed in Table 2. According to the New York Heart Association (NYHA), the patients had different NYHA functional class. Of the 35 patients, 13 patients (37.1%) had LV systolic dysfunction (LVEF< 50%), and 22 patients (62.9%) with LVEF≥50% before TAVI.

**Table 2:** Baseline clinical characteristic of enrolled 35 patients

Demographic data	
Age^a^ (year)	79.6 ± 4.3 (72–87)
Female^b^ n (%)	15 (42.9)
BMI^a^ (kg/m^2^)	22.9 ± 3.72
Body surface area^a^ (m^2^)	1.68 ± 0.18
Heart rate^a^ (beats/min)	69.1 ± 10.8
Systolic BP^a^ (mmHg±SD)	134.5 ± 2.9
Diastolic BP^a^ (mmHg±SD)	65.7 ± 11.5
Hemoglobin^a^ (g/L ± SD)	114.6 ± 11.3
Creatinine^a^ (mmol/L ± SD)	81.5 ± 21.3
STS score^a^ (%mortality±SD)	8.8 ± 4.2
EuroSCORE^a^	3.6 ± 1.9
Artificial aortic valve^b^ n (%)	
Medtronic Hancock II	1(2.9)
VENUS-A	23 (65.7)
J-Valve	11 (31.4)
Medical history^b^ n (%)	
Hypertension	17 (48.6)
Diabetes	9(25.7)
Coronary heart disease (CHD)	14 (40)
Previous PCI	3 (8.5)
COPD	6 (17.1)
Degree of mitral regurgitation^b^ n (%)	
None/Mild	22 (62.9)/13 (37.1)
Mitral annular calcification n (%)	17 (48.6)
NYHA functional classification^b^ n (%)	
I/II/III/IV	0/8 (22.9)/24 (68.6)/3 (8.5)
Diastolic dysfunction^b^ n (%)	
Normal/I/II/ III/Indeterminate	7 (20)/7 (20)/14 (40)/5 (14)/2 (6)

*BP* blood pressure; *STS* society of thoracic surgeons; *PCI* percutaneous coronary intervention; *COPD* chronic obstructive pulmonary disease; *NYHA* New York Heart Association;

^a^Data are mean ± SD with range in parentheses

^b^Data are raw number with percentage in parentheses

Baseline echocardiographic characteristics of the study population were showed in Table 3. After TAVI, an improvement in LVEF was observed with a significant reduction of LV dimension (LVEDD, LVESD) and LV mass index (LVMI). Peak and mean transaortic pressure gradient and maximal aortic jet velocity were significantly decreased.

**Table 3:** Baseline and follow-up echocardiographic variables, pre- and post-TAVI

	Baseline	Post-3d	Post-1 m	Post-6 m	*P*1	*P*2	*P*3
LVEDD (mm)	51.7 ± 7.2	50.6 ± 6.3	50.6 ± 6.4	48.9 ± 5.0	0.336	0.143	0.004^*^
LVESD (mm)	36.4 ± 8.0	36.0 ± 8.4	35.7 ± 5.3	33.3 ± 4.1	0.669	0.604	0.008^*^
IVST (mm)	13.4 ± 1.6	12.8 ± 2.2	12.7 ± 1.7	11.9 ± 1.3	0.028^*^	0.014^*^	0.001^*^
LVPWT (mm)	12.3 ± 0.8	11.5 ± 1.3	11.7 ± 1.1	11.1 ± 1.3	0.002^*^	0.080	0.003^*^
LVEF-2D (%)	52.5 ± 10.1	56.7 ± 12.8	58.7 ± 7.3	61.5 ± 6.6	0.014^*^	0.001^*^	< 0.001^*^
LVMI (g/m^2^)	103.9 ± 12.5	103 ± 12.3	91.5 ± 11.9	84.3 ± 10.7	0.667	< 0.001^*^	< 0.001^*^
Peak VG (mmHg)	102.8 ± 31.1	21.9 ± 8.9	24.7 ± 7.4	21.5 ± 7.4	< 0.001^*^	< 0.001^*^	< 0.001^*^
Mean VG (mmHg)	58.4 ± 16.8	11.6 ± 4.5	12.4 ± 3.8	10.8 ± 3.9	< 0.001^*^	< 0.001^*^	< 0.001^*^
Peak velocity (m/s)	4.9 ± 0.6	2.2 ± 0.5	2.4 ± 0.3	2.2 ± 0.4	< 0.001^*^	< 0.001^*^	< 0.001^*^
SPAP (mmHg)	46.5 ± 14.6	38.5 ± 9.5	37.1 ± 8.6	36.6 ± 6.2	0.022^*^	0.003^*^	0.004^*^

*LVEDD* left ventricular end-diastolic diameter; *LVESD* left ventricular end-systolic diameter; *IVST* interventricular septum thickness; *LVPWT* left ventricular posterior wall thickness; *LVMI* left ventricular mass index; *VG* valve gradient; *SPAP* systolic pulmonary arterial pressure

Data are mean ± SD

*P*1: for comparison between baseline and Post-3d; *P*2: for comparison between baseline and Post-1 m; *P*3: for comparison between baseline and Post-6 m. ^*^Data are *P* < 0.05

### Echocardiographic diastolic parameters characteristics

**Fig. 2:**
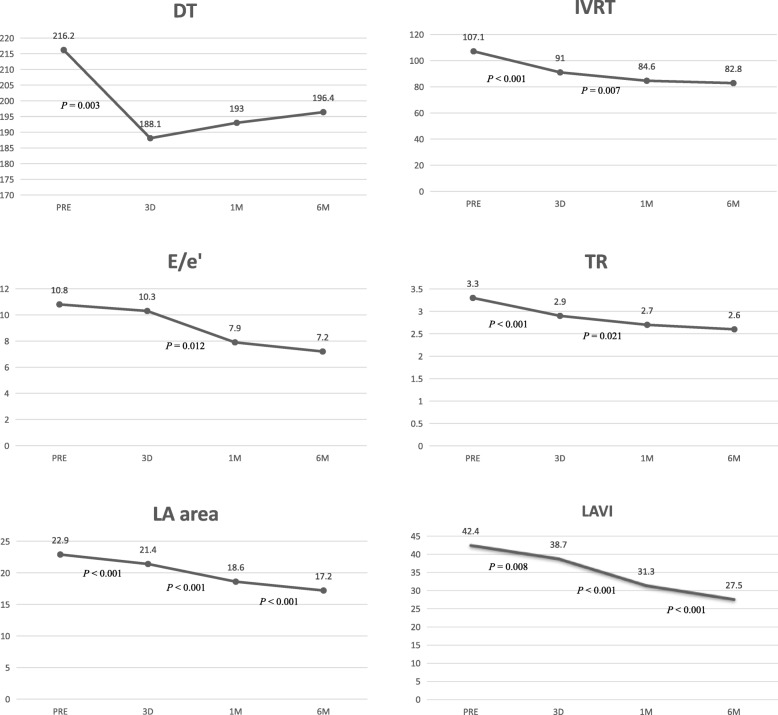
broken line graphs show the distributions and variation of E/e’, TR, LA area, LAVI, IVRT and DT on one day before TAVI and on the third day, in the first-, and six- month after TAVI

Mitral inflow and DTI parameters, as well as LA size were summarized in Table 4. Before TAVI, patients with diastolic dysfunction had lower E, E/A, E’ and higher A, IVRT, E/E’ and LAVI than the normal range. After TAVI, the parameters were significantly changed compared with the previous exam (PRE vs.3D; 3D vs.1 M; 1 M vs. 6 M) (Fig. 2). Improvement of IVRT, TR and DT occurred immediately after TAVI, while DT remained reduced until the third after surgery and IVRT and TR remained significantly reduced until 1-month after surgery. Improvements in E/e’ and e’ occurred later; E/e’ was reduced by 1-month while e’ increased by 6-months. LA area and LAVI presented a statistical drop for six straight months. No significant difference was found in E, A, or E/A(all *P*>0.05) over time.

**Table 4:** Echocardiographic diastolic variables, pre- and post-TAVI

	Baseline	Post-3d	Post-1 m	Post-6 m	*P*1	*P*2	*P*3
E (cm/s)	66.3 ± 18.6	67.7 ± 16.9	66.0 ± 20.8	66.5 ± 15.8	0.582	0.561	0.850
A (cm/s)	81.4 ± 22.7	86.9 ± 26.7	87.5 ± 23.2	85.5 ± 20.4	0.072	0.882	0.585
E/A	0.9 ± 0.4	0.8 ± 0.4	0.8 ± 0.3	0.9 ± 0.3	0.959	0.491	0.896
DT (ms)	216.2 ± 50.6	188.1 ± 39.3	193.0 ± 34.0	196.4 ± 26.4	0.003^*^	0.540	0.514
IVRT(ms)	107.1 ± 12.1	91.0 ± 12.3	84.6 ± 9.1	82.8 ± 9.3	< 0.001^*^	0.007^*^	0.273
E’ (cm/s)	6.5 ± 2.1	6.9 ± 1.8	8.0 ± 2.3	8.6 ± 1.6	0.356	0.018^*^	0.047^*^
E/E’	10.8 ± 3.7	10.3 ± 3.3	7.9 ± 3.0	7.2 ± 2.1	0.403	0.012^*^	0.063
LA area (cm^2^)	22.9 ± 4.6	21.4 ± 3.8	18.6 ± 3.0	17.2 ± 2.3	< 0.001^*^	< 0.001^*^	< 0.001^*^
LAVI (ml/cm^2^)	42.4 ± 14.7	38.7 ± 11.3	31.3 ± 8.3	27.5 ± 5.5	0.008^*^	< 0.001^*^	< 0.001^*^
TR (m/s)	3.3 ± 0.5	2.9 ± 0.5	2.7 ± 0.4	2.6 ± 0.3	< 0.001^*^	0.021^*^	0.143

E, early transmitral flow velocity; A, late transmitral flow velocity; *DT* deceleration time of E-wave; *IVRT* isovolumic relaxation time; E′, mean peak early diastolic myocardial annular velocity; *TR* tricuspid regurgitation systolic peak velocity; *LA* left atrial; *LAVI* left atria maximum volume index

Data are mean ± SD

*P*1: for comparison between baseline and Post-3d; *P*2: for comparison between Post-3d and Post-1 m; *P*3: for comparison between Post-1 m and Post-6 m. ^*^Data are *P* < 0.05

### Diastolic functional grading evaluations

**Fig. 3:**
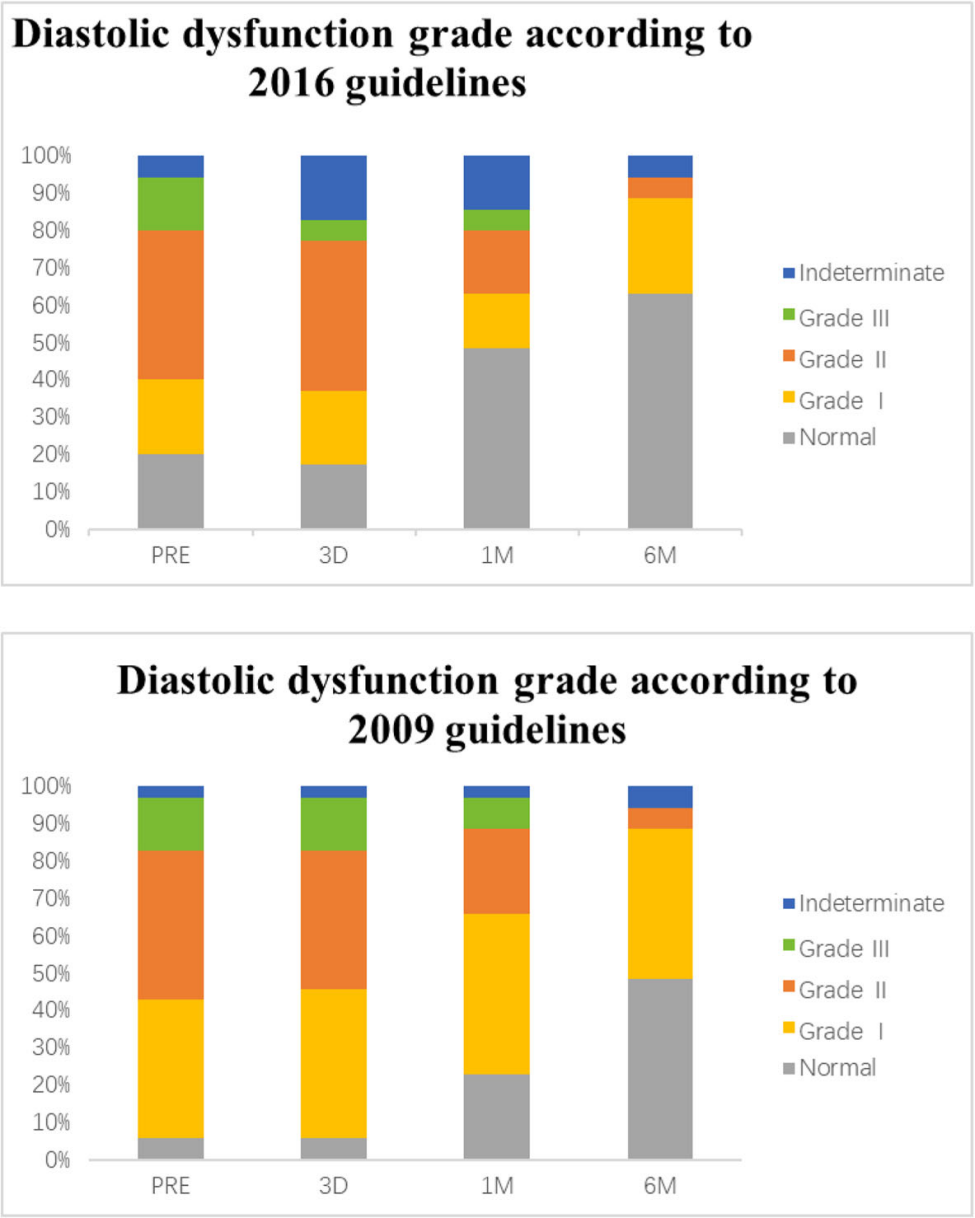
Bar graphs show the distributions of LVDD grading according to 2009 ASE/ EAE and 2016 ASE/EACVI recommendations

**Fig. 4:**
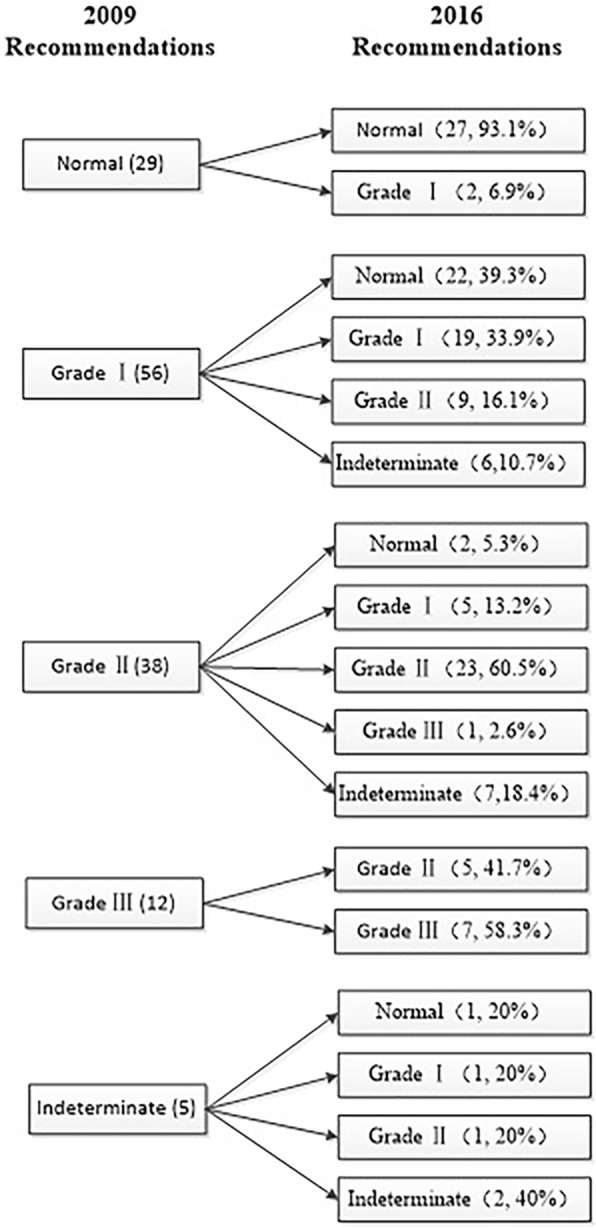
Changes in LVDD groups after the reevaluation between the 2009 ASE/EAE recommendations (left) and the 2016 ASE/EACVI recommendations (right)

With the 2009 guidelines, the number of patients at each grade changed over time [Normal: 2 (5.7%) VS 2 (5.7%); 8 (22.9%); 17 (48.6%); I: 13 (37.1%) VS 14 (40%); 15 (42.3%); 14 (40%); II:14 (40%) VS 13 (37.1%); 8 (22.9%); 2 (5.7%)); III: 5 (14.3%) VS 5 (14.3%); 3 (9%); 0 (0%)]. For indeterminate, there was one patient (2.9%) before TAVI, 1 (2.9%) on the third day, 1 (2.9%) in the first month, and there were 2 (5.7%) in the sixth month in dysfunction classification. When using the new 2016 guidelines, the change was showed as the following [Normal: 7 (20%) VS 6 (17%); 17 (49%); 22 (62%); I: 7 (20%) VS 7 (20%); 5 (14%); 9 (26%); II: 14 (40%) VS 14 (40%); 6 (17%); 2 (6%)); III: 5 (14%) VS 2 (6%); 2 (6%); 0 (0%)]. For indeterminate, there were two patients (6%) before TAVI, 6 (17%) on the third day, 5 (14%) in the first month, and 2 (6%) in the sixth month (Fig. 3). For comparing with two guidelines, we respectively evaluated four times for each patient and totally calculated 140 times. Of 140 times assessment, 62 times (44%) by the 2009 recommendations were reclassified with different grades when using 2016 guidelines and these changes were showed detailedly in Fig. 4. Specifically, 39.3% of individuals initially classified as grade I LVDD by 2009 recommendations were reclassified with normal diastolic function when using 2016 guidelines. Comparing PRE and 6 M, according to 2009 guidelines, 19 patients improved 1 grade, 8 patients improved 2 grades; while according to 2016 guidelines, 9 patients improved 1 grade, 13 patients improved 2 grades,1 patient improved 3 grades.

Of the 35 patients, 9 patients had EF less than 50% preoperatively, and 8 had EF increased to more than 50% at different time points after TAVI (three patients improved in post 3 days, 3 improved in post 1 month, 2 improved in post 6 month). These patients were evaluated separately using two guidelines. Except that there was no difference in two patients, 2009 guidelines have still overestimated diastolic dysfunction grading in 5 patients (from grade III to II, or from grade I to Normal). Besides, there was just one patient be underestimated (from grade I to II).

**Fig. 5:**
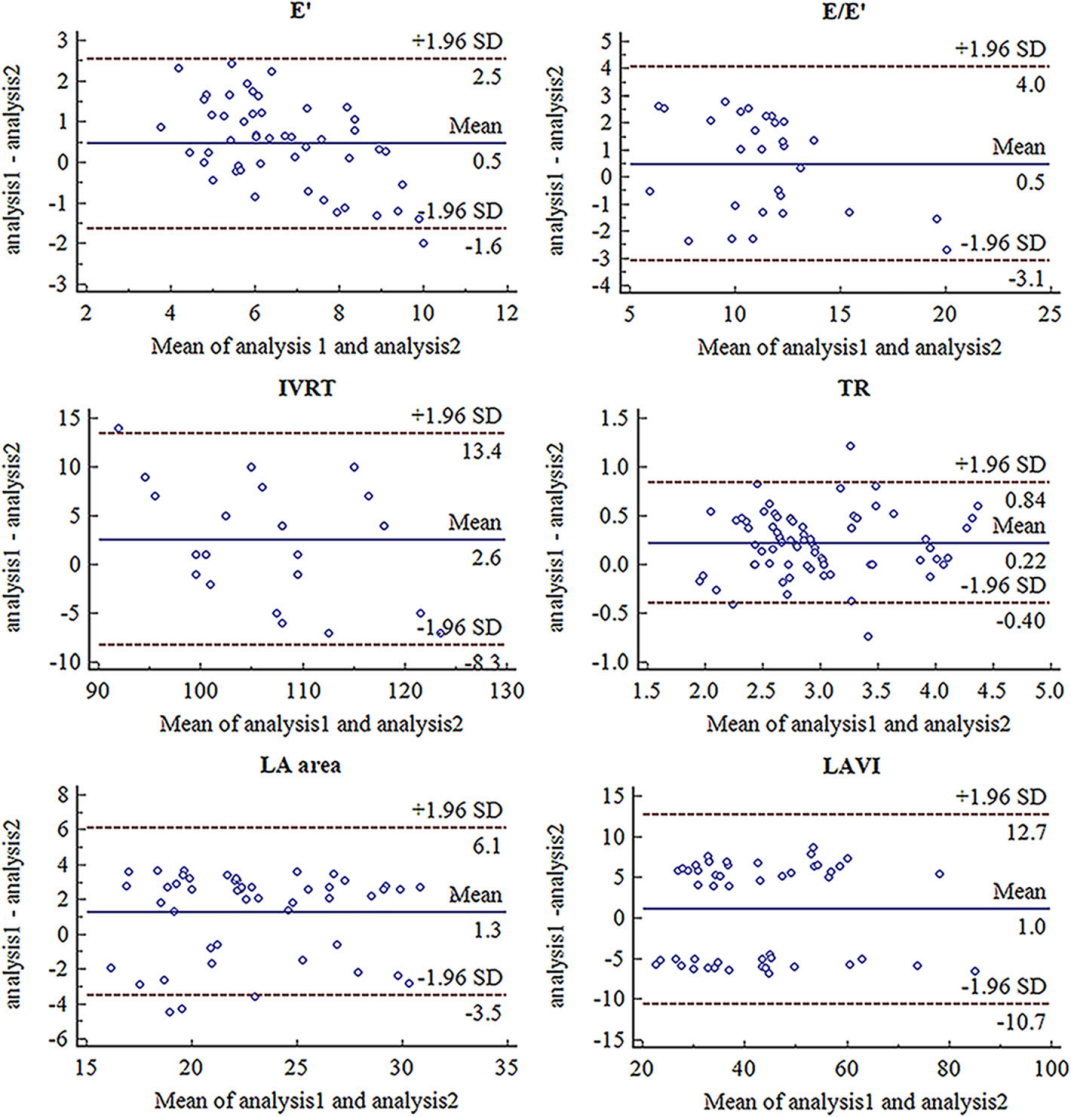
The Bland-Altman analysis of E’, E/e’, IVRT, TR, LA area and LAVI

The ICC for IVRT was 0.799 (95% CI: 0.616–0.901), for E’ 0.803 (95% CI: 0.675–0.883), for E/E’ 0.846 (95% CI: 0.701–0.925), for TR 0.875 (95% CI: 0.822–0.913), for LA area 0.846 (95% CI: 0.743–0.910), for LAVI 0.916 (95% CI: 0.856–0.952). The Bland-Altman analysis were in Fig. 5, indicating good reliability of these diastolic functional parameters.

## Discussion

In this study, we used conventional 2D transthoracic echocardiography and Doppler imaging to display the dynamic improvement process of LV diastolic parameters within 6 months after TAVI in AS patients and evaluated LVDD separately with 2009 and 2016 recommendations. The results showed: (1) 2D echocardiography and Doppler imaging could effectively reflect the change of LVDF by echocardiographic parameters in AS patients after TAVI. (2) For LVDD classification, the updated 2016 ASE/ EACVI recommendations simplified the approach and revealed obvious differences from the 2009 guidelines. Specifically, more patients experienced an improvement in grading and can be regarded as benefiting from TAVI according to 2016 recommendations.

Understanding the dynamic process may be helpful for clinical relevance, for example, clinicians can predict the postoperative trends by analyzing echocardiographic data and convey valuable information to patients. E/e’ is an excellent indicator of left ventricular end diastolic pressure (LVEDP) and increasing in E/e’ suggest elevated LV filling pressures [16, 17]. Sari et al. reported that E/e’ represented no obvious change within 24 h after TAVI, and a significant decrease was found after the first month in the results of Blair et al. [18, 19]. Our results agreed with these findings and then went a step further. E/e’ had obvious improvement from the third day to first month, and remained stable until the sixth months—in particular, this fact exactly reflected the dynamic process of LVEDP. We found that the significant variation time range of mean e’ was from the third day to sixth month, which consistent with the results of Vizzardi et al. [20], suggesting that recovering the LV relaxation need to take a long time. LAVI decreased immediately after TAVI and continually until the sixth month. This phenomenon mainly resulted from rapid morphological changes after TAVI.

In our study, nearly half of LVDD assessments were inconsistent with 2009 and 2016 recommendations. Specifically, a substantial fraction of individuals initially classified as grade I LVDD by 2009 guidelines were reclassified with normal diastolic function by 2016 recommendations. In other words, this data revealed an overestimation of grade I when using 2009 recommendations. One important reason for the overestimating may be caused by the remove of Valsalva E/A from 2016 guidelines. The Valsalva maneuver made E/A reduced. Patients must generate and sustain a sufficient increase in intrathoracic pressure, and the examiner needed to maintain the correct sample volume location between the mitral leaflet tips during the maneuver [11]. If patients performed this maneuver improperly, E/A could be overestimated. On the other hand, TR as an indirect index to reflect left atrial pressure (LAP), was a new indicator in 2016 recommendations. Due to the transfer process of the pressure, the increase of TR has occurred later than LAP, which may raise the threshold for elevating LAP, and this further avoided the overestimation of LVDD. In addition, we also found that 2009 guidelines underestimated two cases who had slightly reduced LVEF and normal LVDF. According to 2016 recommendations, patients with decreased EF would be classified into at least grade I LVDD. This was consistent with the theory that LVDD occurred earlier than systolic dysfunction [21, 22].

We admit that there were some limitations to our study. First, the study was a single centre and retrospective study, and our sample was relatively small. We look forward to external data to validate our results. Second, there were 15 patients with mitral annular calcification (MAC). Although the report did not indicate severity degree, according to 2016 recommendation, in patients with moderate to severe mitral annular calcification, the lateral e′ may be decreased due to restriction of the posterior mitral leaflet excursion [23]. Thus, an increase in lateral E/e′ occured due to the mechanical effect of calcification. Therefore, separation of the effect of MAC from that of LV diastolic dysfunction on lateral E/e′ ratio may not be possible in the individual patient. Since no further study was indicated for the influence of calcification on septal e′, we used lateral e′, septal e′, and average E/e′ value to assess diastolic function together, which may reduce the impact of MAC. Third, we did not included BNP levels and cardiovascular outcomes, and further analyze the relationship between LVDD classification and clinical outcomes.

## Conclusion

To summarise, the conventional echocardiography and Doppler imaging were useful methods to reflect variation process of LVDF in AS patients after TAVI. And we also demonstrated that there were obvious differences in LVDD classification between the 2009 guidelines and updated 2016 ASE/ EACVI recommendations. Specifically, more patients experienced an improvement in grading and can be regarded as benefiting from TAVI by 2016 recommendations. In the future, the validation and improvement of our findings in a larger sample and in other institutions are warranted.

## Data Availability

The datasets generated and/or analysed during the current study are not publicly available due [REASON WHY DATA ARE NOT PUBLIC] but are available from the corresponding author on reasonable request.

## Abbreviations

AS: Aortic stenosis
DT: E wave deceleration time
ICC: Intraclass correlation coefficient
IVRT: Isovolumic relaxation time
LAP: Left atrial pressure
LVDD: Left ventricular diastolic dysfunction
LVDF: Left ventricular diastolic function
LVEDP: Left ventricular end diastolic pressure
PW: Pulsed-wave
SAVR: Surgical aortic valve replacement
TAVI: Transcatheter aortic valve implantation
TDI: Tissue Doppler imaging
TR: Systolic tricuspid regurgitation velocity
TTE: Transthoracic echocardiography
